# Flow cytometric analysis of immune cell populations in the bronchial and mesenteric lymph nodes of the dromedary camel

**DOI:** 10.3389/fvets.2024.1365319

**Published:** 2024-04-30

**Authors:** Jamal Hussen, Hind Althagafi, Mohammed Ali Al-Sukruwah, Baraa Falemban, Aimi Syamima Abdul Manap

**Affiliations:** ^1^Department of Microbiology and Parasitology, College of Veterinary Medicine, King Faisal University, Al-Ahsa, Saudi Arabia; ^2^Department of Biology, College of Science, Princess Nourah bint Abdulrahman University, Riyadh, Saudi Arabia; ^3^Department of Biomedical Sciences, College of Veterinary Medicine, King Faisal University, Al-Ahsa, Saudi Arabia

**Keywords:** camel, immune system, lymph node, B cells, T cells, flow cytometry

## Abstract

Dromedary camel is an important livestock species with special economic value in arid and semi-arid regions of the world. Given the limited data on detailed immune cell composition and cell marker expression in the dromedary camel lymph node tissue, the present study was undertaken to investigate the immune cell composition of bronchial and mesenteric lymph nodes from healthy dromedary camels using flow cytometry. In this study, we applied flow cytometry and multicolor immuno-fluorescence to phenotype the main populations of immune cells in the bronchial and mesenteric camel lymph nodes and compared them with separated peripheral blood mononuclear cells and granulocytes. We used antibodies to detect several cell surface molecules associated with camel T cells (CD4, WC1), B cells (MHCII, BAQ44A), monocytes/macrophages (CD172a, CD14, CD163), in addition to the pan-leukocyte marker CD45 and the cell adhesion molecules CD44 and CD18. Compared to blood mononuclear cells, camel lymph node cells contained a higher percentage of lymphoid cells with only a minor fraction of myeloid cells. In addition, the lower expression of CD44 and CD18 on lymph node lymphocytes compared to lymphocytes from peripheral blood indicates higher frequency of naïve lymphocytes in the lymph nodes. The frequency of CD4^+^ T cells, B cells and γδ T cells within camel lymph node lymphocytes compared to blood indicates a similar tissue distribution pattern of lymphocyte subsets in camel and bovine and supports previous reports on the similarity between the camel immune system and the immune system of other ruminants. Lymph node neutrophils were identified as CD45^++^ CD172a^++^, CD14^+^, MHCII^low^, BAQ44A^+^, CD44^++^, CD18^++^ cells. In conclusion, the present study is describing the employment of flow cytometric single-cell analysis and immunostaining for the analysis of the immune cell composition in the camel lymph node.

## 1 Introduction

The classification of lymphoid organs encompasses primary organs, including the bone marrow and thymus, where immune cells are generated and undergo differentiation into functional cells. Additionally, secondary organs such as the spleen, lymph nodes, and the mucosa-associated lymphoid organs (MALT) serve as the sites where immune responses occur (1). Lymph nodes are specialized structures dispersed throughout the body and are responsible for the filtration of the lymph (2). They lymph node is traditionally classified into three distinct histological regions, namely the cortex, paracortex, and medulla, with a capsule that surround the node. The lymphatic tissue within the node is characterized by clusters of immune cells, predominantly consisting of lymphoid cells alongside some myeloid cells, such as macrophages and dendritic cells. Within the node tissue compartments, B and T cells home to distinct areas, where they interact with antigen presenting cells and undergo clonal expansion.

The local immune cell composition within a lymphoid organ is indicative of the balance between cellular migration towards the organ and the rates at which immune cells become trapped and released within it during an immune response (3). The analysis of changes in immune cell distribution in different primary and secondary immune organs has been proven a valuable tool to investigate the immune response to infection or vaccination (4, 5). Different techniques were employed for the analysis of immune cell composition of lymph nodes. Techniques like immunohistochemistry are the best choice when analyzing the area-specific cellular composition of the different compartments of the nodes. Flow cytometry in combination with monoclonal antibody (mAb) staining enables single cell analysis that has proven a useful tool to detect and characterize immune cells in cell suspension. It has the advantage of higher sensitivity for the identification in minor changes in cellular composition, which is pivotal in the early diagnosis of pathologies. The applicability of this method has been addressed in areas of immunopathology and tumor research and diagnosis by the identification of the pattern of distribution of a cell population within tissue homogenates (6). In addition, the comparison between flow cytometry and image cytometry revealed that measurements of lymph node cell populations obtained with the two methods are comparable (6).

Flow cytometric analysis of the cellular composition of bovine lymph nodes revealed the dominance of lymphocytes with minor proportions of CD172a-positive cells, including macrophages and neutrophils (7). Although they are mainly recognized as circulatory short-lived innate immune cells with the ability to infiltrate tissues upon infection or inflammation, neutrophils are now discussed as bridging cells at the interface of innate and adaptive immune systems (8). Recent studies reported the presence of tissue-resident neutrophils in the lymph node under steady-state conditions before the onset of inflammation (9). They enter uninfected lymph nodes via high endothelial venules and patrol the lymph nodes of the skin and mucosal systems under homeostatic conditions (10).

Although some immunohistochemical studies described the structure of the camel lymph nodes and identified some immune cells with a distribution that seems similar to the bovine lymph node (11, 12), flow cytometric analysis of the immune cell composition of the camel lymph node has not been investigated so far. Therefore, the present study was undertaken to test the reactivity of camel lymph node cells with monoclonal antibodies to selected cell surface antigens using flow cytometry. Uncovering the immune cell composition of lymph nodes in healthy animals will help in the establishment of normal values that could be used as reference values for the diagnosis of immunopathology and the analysis of local immune response in lymphoid organs to infection and vaccination.

## 2 Materials and methods

### 2.1 Animals and collection of blood and lymph nodes samples

Ten healthy male one-humped camels (*Camelus dromedarius*) were included in the present study for the collection of blood (only sex animals) and lymph node samples (ten animals). The animals (aged between 8 and 11 years; mean age 8.9 ± 1.1 years) were selected from the camels admitted for normal slaughtering for human consumption at Al-Omran Slaughterhouse in Al-Ahsa Region in Saudi Arabia. All the camels were from Al-Majaheem breed. Each animal was clinically examined for clinical signs like injuries, reproductive, gastrointestinal, or respiratory disease. Venous blood was collected from the jugular vein into vacutainer tubes containing EDTA (Becton Dickinson, Heidelberg, Germany). Directly after euthanasia of animals via bleeding, all viscera were examined to exclude animals with signs of abdominal, thoracic, or reproductive disorders. After collection, bronchial and mesenteric lymph nodes were immediately placed on ice in cold PBS containing 1 mM EDTA and transported to the laboratory within one hour.

### 2.2 Separation of blood mononuclear cells and neutrophilic granulocytes

Peripheral blood mononuclear cells (MNC) were separated as previously described (13). Briefly, 10 mL camel blood was diluted 1:2 in PBS and overlaid on 15 mL Lymphoprep^™^ (STEMCELL Technologies Inc. Vancouver, BC, Canada). After centrifugation for 30 min at 1000 × g and 10°C without brake, the MNC were collected from the interphase into new 15 mL tubes containing 2 mL cold PBS. The collected cells were washed three times with PBS (500, 250, and 100 × g, each for 10 min). Finally, the cell pellet was resuspended in PBS and adjusted to 1 × 10^7^ cells/mL. Cell viability (always more than 95%) was evaluated by flow cytometry after adding propidium iodide (2 μg/mL) to the cells. The immunophenotype of blood neutrophils was analyzed by staining whole blood leukocytes. For this, red blood cells were removed by hypotonic lysis and the remaining cell pellet were suspended in PBS (5 × 10^6^ cells/mL).

### 2.3 Cell separation from lymph node samples

Bronchial and mesenteric lymph node cell suspension was prepared as previously described (14) with some modifications. Firstly, fat and connective tissue were removed from the capsule and the lymph nodes were placed in sterile Petri dishes containing cold PBS-EDTA. The nodes were then cut into small pieces and minced using sterile scissors and forceps. The minced lymph nodes were suspended in PBS-EDTA and the cell supernatants were passed through a cell strainer. After that, the cell suspensions prepared from different regional pieces were merged to homogenize the content making it representative for the whole lymph node. After a washing step in PBS-EDTA for 10 min at 4°C and 300 × g, the cell pellet was resuspended in PBS-EDTA and contaminating red blood cells were removed by hypotonic lysis. Finally, the cell pellet was resuspended in PBS-EDTA at 1.0 × 10^7^ cells/mL. Cell viability (always more than 95%) was evaluated by flow cytometry after adding propidium iodide (2 μg/mL) to the cells.

### 2.4 Antibodies used in the study

The specificity, clone, species of origin, and commercial provider for all primary and secondary antibodies used in the current study are described in Table 1. The antibody combinations used for the multi-color staining are described in Table 2.

**Table 1:** List of antibodies.

Antigen	Antibody clone	Labeling	Source	Isotype
CD45	LT12A	–	Kingfisher	Mouse IgG2a
CD44	LT41A	–	Kingfisher	Mouse IgG2a
CD14	CAM36A	–	Kingfisher	Mouse IgG1
MHCII	TH81A5	–	Kingfisher	Mouse IgG2a
CD172a	DH59b		Kingfisher	Mouse IgG1
CD163	LND68A	–	Kingfisher	Mouse IgG1
CD4	GC50A1	–	VMRD	Mouse IgM
WC1	BAQ128A	–	VMRD	Mouse IgG1
B cells	BAQ44A	–	Kingfisher	Mouse IgM
CD18	6.7	FITC	BD	Mouse IgG2a
Mouse IgM	poly	APC	Thermofisher	Goat IgG
Mouse IgG1	poly	FITC	Thermofisher	Goat IgG
Mouse IgG2a	poly	PE	Thermofisher	Goat IgG

MHC, major histocompatibility complex; WC1, workshopcluster 1; APC, allophycocyanin; FITC, fluorescein isothiocyanate; PE, phycoerythrin; poly, polyclonal.

**Table 2:** Staining combinations.

Combination	Primary antibody cocktail	Secondary antibody cocktail
1	CD45/CD172a	Mouse IgG1-FITC/Mouse IgG2a-PE
2	CD4/WC1	Mouse IgG1-FITC/Mouse IgM-APC
3	CD14/MHCII/BAQ44A	Mouse IgG1-FITC/Mouse IgG2a-PE/Mouse IgM-APC
4	CD163/CD44	Mouse IgG1-FITC/Mouse IgG2a-PE
5	CD18-FITC	
6	CD4/MHCII	Mouse IgG2a-PE/Mouse IgM-APC
7	–	Mouse IgG1-FITC/Mouse IgG2a-PE/Mouse IgM-APC

MHC, major histocompatibility complex; WC1, workshop cluster 1; APC, allophycocyanin; FITC, fluorescein isothiocyanate; PE, phycoerythrin.

### 2.5 Membrane immunofluorescence and flow cytometry

Lymph node cells, blood MNC, or total leukocytes were labeled with monoclonal antibodies (mAbs) to camel leukocyte antigens and analyzed by flow cytometry (13). Separated cells (1 × 10^6^ cells/well) were incubated in the wells of a 96-well plate with mAbs cross-reactive with the homologous camel molecules: CD45, CD44, BAQ44A, WC1, CD4, CD18, CD172a, CD14, CD163, and MHCII (15, 16). After incubation (15 min; 4°C), cells were washed twice and were incubated with mouse secondary antibodies (IgM, IgG1, IgG2a; Invitrogen) labelled with different fluorochromes or with mouse isotype control antibodies (Becton Dickinson Biosciences). After washing, cells were analyzed on a Becton Dickinson Accuri C6 flow cytometer (Becton Dickinson Biosciences, San Jose, California, United States). Data of at least 100,000 cells were collected and analyzed with the flow cytometric software C-Flow (Becton Dickinson Biosciences, San Jose, California, United States). For multi-color staining tubes, correction of fluorescence spillover was done after calculation of compensation matrix using the Accuri C6 compensation calculator Excel sheet. For this, a set of single-stained control samples was used to determine the extent of fluorescence spillover from each fluorochrome.

### 2.6 Statistical analyses

Data analysis was performed using the flow cytometric software C-Flow (BD). Means and standard error of mean (SEM) were calculated using the column statistic function of the Prism software (GraphPad). Differences between means were tested with *t*-test with *p*-value of less than 0.05 being considered significant.

## 3 Results

### 3.1 Lymph node and peripheral blood mononuclear cells show different fractions of lymphocytes and monocytes

**Figure 1:**
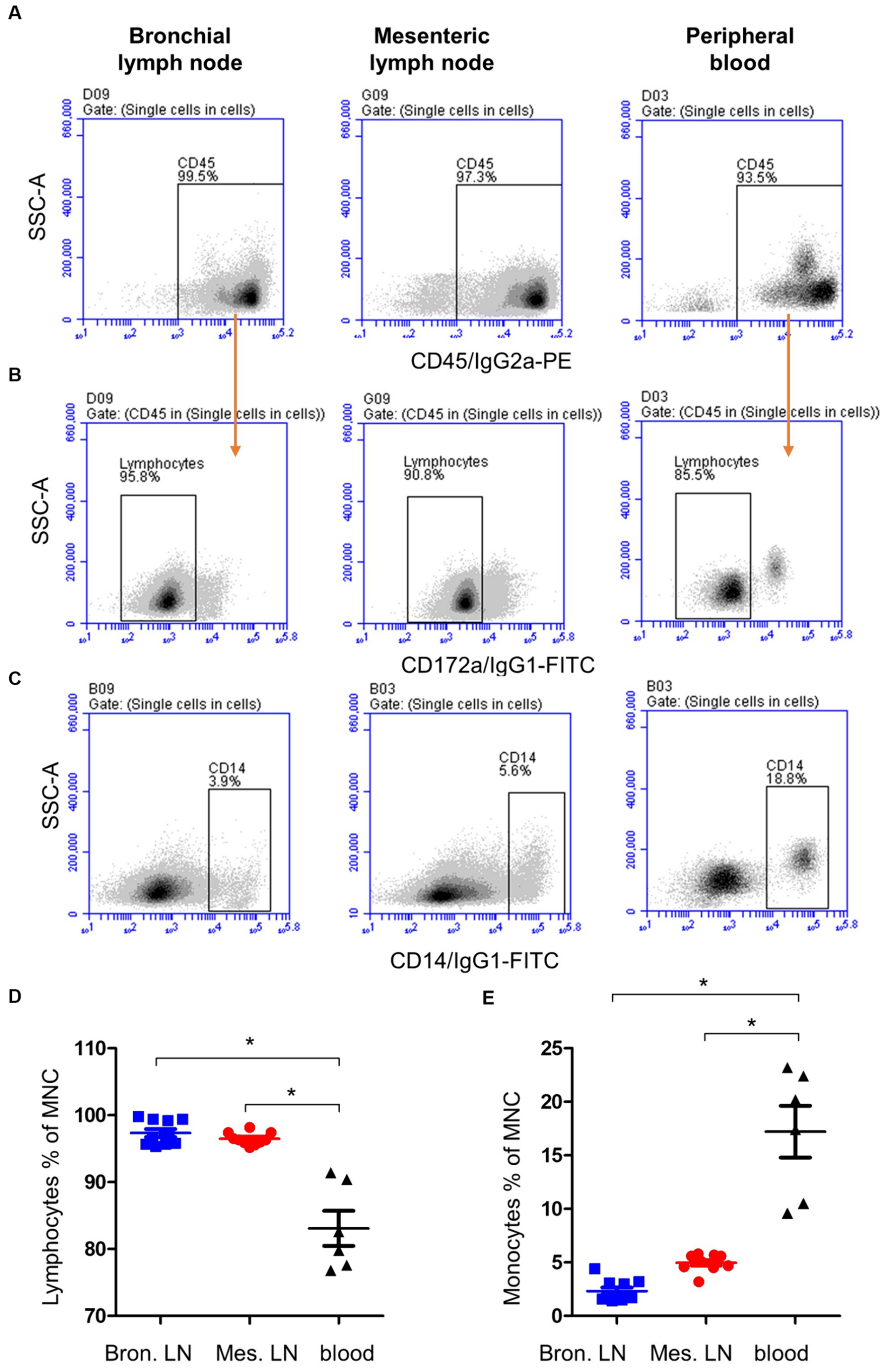
Gating strategy for the flow cytometric analysis of blood and lymph node (bronchial and mesenteric) mononuclear cells. Cells were stained with mAbs to CD45, CD172a, and CD14 and analyzed on the flow cytometer. **(A)** The percentage of cells stained positive with CD45 mAb was identified within single cells after excluding the cell doublets. **(B)** Staining of lymph node cells and blood MNC with mAbs to CD172a within CD45-positive cells. **(C)** Staining pattern of lymph node cells and blood MNC with mAbs to CD14. The percentage of lymphocytes **(D)** and monocytes **(E)** of total bronchial (blue color), mesenteric (red color) lymph node, or blood MNC (black color) were calculated and presented. * indicates significant differences (*n* = 10 animals for lymph node samples and six animals for blood samples; *p* < 0.05).

The majority of both lymph node cells and blood MNC stained positively with mAbs to the pan-leukocyte marker CD45 (Figure 1A). A higher (*p* < 0.05) fraction of lymphocytes (CD172a− CD45+) was found within bronchial (97.9 ± 0.2; mean ± SEM) and mesenteric (96.6 ± 0.5) lymph node than blood MNC (83.1 ± 2.6) (Figures 1B,D). Staining with mAbs to CD14 revealed a lower percentage of monocytes within bronchial (2.3 ± 0.2) and mesenteric (5.2 ± 0.2) lymph node cells compared to blood (17.2 ± 2.4) MNC (Figures 1C,E). The complete gating strategy is described in the Supplementary Figure S1.

### 3.2 Different cellular composition of lymph node and blood lymphocytes

**Figure 2:**
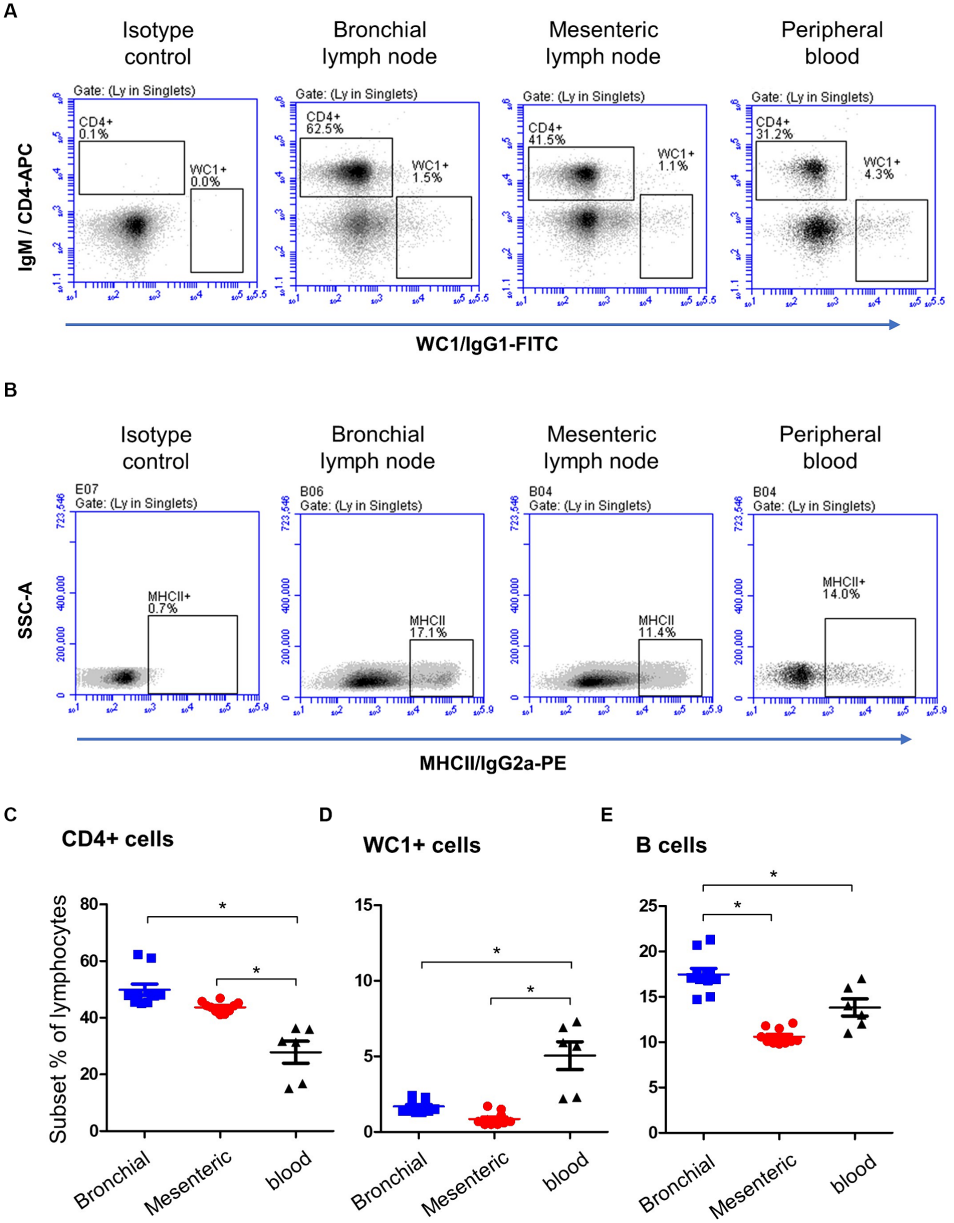
Lymphocyte subsets in bronchial and mesenteric lymph node and blood of camels. **(A)** After gating on lymphocytes within MNC, CD4+ and WC1+ cells were identified based on their staining with corresponding monoclonal antibody compared to isotype control staining. **(B)** Camel B cells were identified as MHCII-positive cells within the lymphocyte population (CD14-negative MNC). The percentages of CD4+ **(C)**, WC1+ **(D)**, and B cells **(E)** within lymphocytes were calculated and presented comparatively for bronchial (blue color), mesenteric (red color) lymph node, or blood MNC (black color).

Staining of blood and lymph node cells with monoclonal antibodies to camel CD4, WC1 (Figure 2A), and MHCII (Figure 2B), revealed higher (*p* < 0.05) percentage of CD4+ lymphocytes (Figure 2C) in bronchial (49.2 ± 3.3) and mesenteric (43.7 ± 0.9) lymph nodes than in blood (27.8 ± 3.8), while the percentage of WC1+ lymphocytes (Figure 2D) was significantly higher (*p* < 0.05) in blood (5.0 ± 0.2) than bronchial (1.6 ± 0.9) and mesenteric lymph nodes (0.8 ± 0.9). Bronchial lymph node lymphocytes (17.4 ± 0.6) contained the highest (*p* < 0.05) percentage of B cells compared to mesenteric lymph nodes (10 ± 0.3) and blood lymphocytes (13.8 ± 0.9) cells (Figure 2E). The complete gating strategy is described in the Supplementary Figure S2.

### 3.3 The camel B cell marker BAQ44A is differently expressed on lymph node and blood lymphocytes

**Figure 3:**
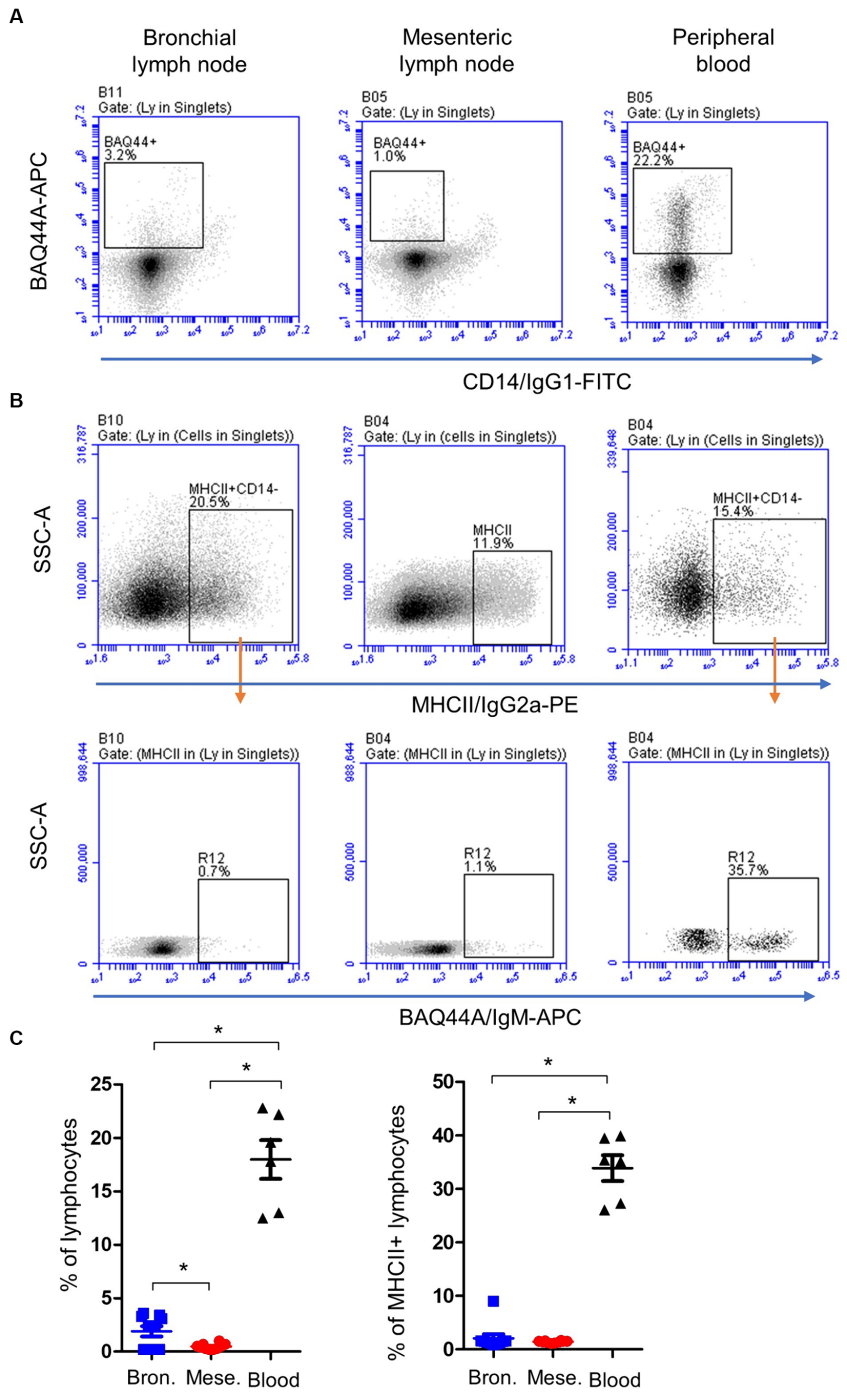
Expression of the B cell marker BAQ44A on bronchial and mesenteric lymph node and blood lymphocytes. Lymph node cells or blood MNC were labeled with antibodies to CD14, MHCII, and BAQ44A. **(A)** The fraction of BAQ44A+ cells within lymphocytes was identified as CD14-BAQ44A+ cells. **(B)** After gating on lymphocytes (CD14- MNC), a gate was placed on MHCII+ cells and the percentage of BAQ44A+ cells within MHCII+ cells was identified in a separate dot plot. **(C)** The BAQ44A+ cells within total lymphocytes or within B cells (MHCII+ lymphocytes) were calculated and presented for bronchial (blue color), mesenteric (red color) lymph node, or blood MNC (black color).

A combined staining with antibodies to CD14, MHCII, and BAQ44A (Figures 3A,B) revealed different expression of BAQ44A on lymph node and blood cells. The percentage of BAQ44A+ cells within total lymphocytes was ten-time higher (*p* < 0.05) in blood (17.9 ± 1.8) than bronchial (1.9 ± 0.2) lymph nodes. The lowest (*p* < 0.05) frequency of BAQ44A+ cells was, however, found in mesenteric lymph nodes (0.4 ± 0.1). Similarly, blood MHCII+ lymphocytes contained a higher (*p* < 0.05) fraction of BAQ44A+ cells than bronchial and mesenteric lymph nodes cells (Figure 3C).

### 3.4 Expression of the cell adhesion molecules CD44 and CD18 on lymph node and blood lymphocytes

**Figure 4:**
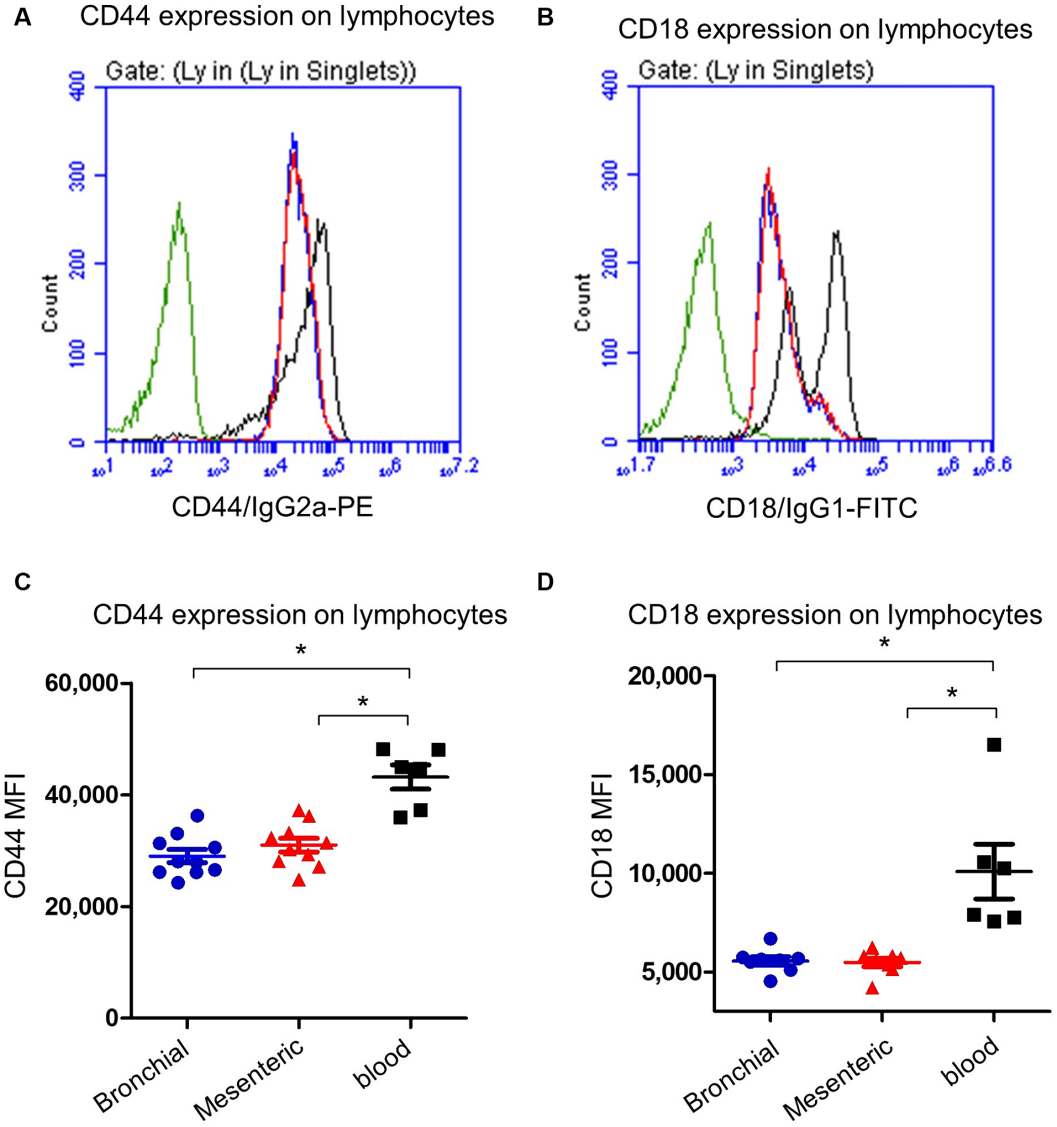
Expression of the cell adhesion molecules CD44 and CD18 on camel lymph node and blood lymphocytes. Camel lymph node cells and blood MNC were stained with antibodies to camel CD44 or CD18 and analyzed by flow cytometry. Histogram presentation of CD44+ **(A)** or CD18+ **(B)** lymphocytes within bronchial lymph node (blue line), mesenteric lymph node (red line), and blood cells (black line) in comparison to isotype control (green line). Mean fluorescence intensity (MFI) of CD44+ cells **(C)** or CD18+ cells **(D)** were calculated and presented in scattered plot graphs for bronchial (blue color), mesenteric (red color) lymph node, or blood MNC (black color).

Bronchial and mesenteric lymph node lymphocytes showed lower expression (*p* < 0.05) of the cell surface molecule CD44 (Figures 4A,C) compared to lymphocytes from peripheral blood with a mean fluorescence intensity (MFI) value cells of 29,472 ± 1,676 on mesenteric lymph node compared to bronchial lymph node (29,047 ± 1,135) and blood cells (43,180 ± 2,174). Similarly, the expression of CD18 was significantly (*p* < 0.05) lower on bronchial (5,563 ± 214) and mesenteric lymph nodes (5,675 ± 102) lymphocytes than blood (10,091 ± 1,393) lymphocytes (Figures 4B,D).

### 3.5 Lymph node and peripheral blood neutrophils showed different immunophenotype

**Figure 5:**
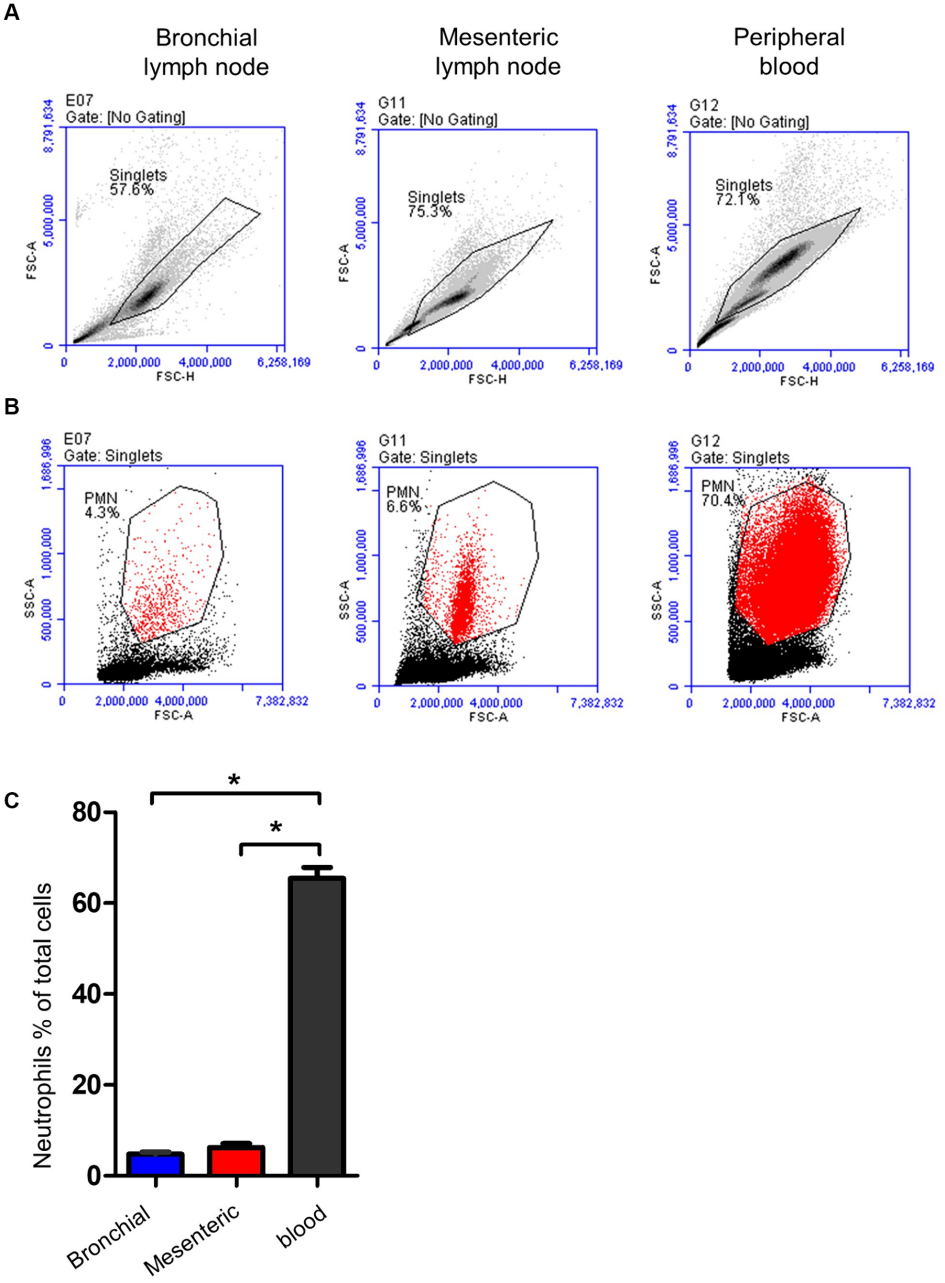
Gating strategy for lymph node and blood neutrophils (PMN). **(A)** Camel leukocytes or lymph node cells were analyzed by flow cytometry. **(B)** Neutrophils were identified based on their forward and side scatter properties and their percentages within bronchial (blue color), mesenteric (red color) lymph node, or blood leukocytes (black color) were presented **(C)**.

Neutrophils were identified within lymph nodes and blood cells based on their light scatter properties (Figures 5A,B). With a mean of 4.7 (±0.4; standard error of the mean; SEM) for bronchial lymph nodes and 6.2 ± 0.8 for mesenteric lymph nodes, neutrophils were only a minor fraction within lymph nodes cells (Figure 5C), which is significantly (*p* < 0.05) lower than their percentage in peripheral blood (72 ± 2.4).

**Figure 6:**
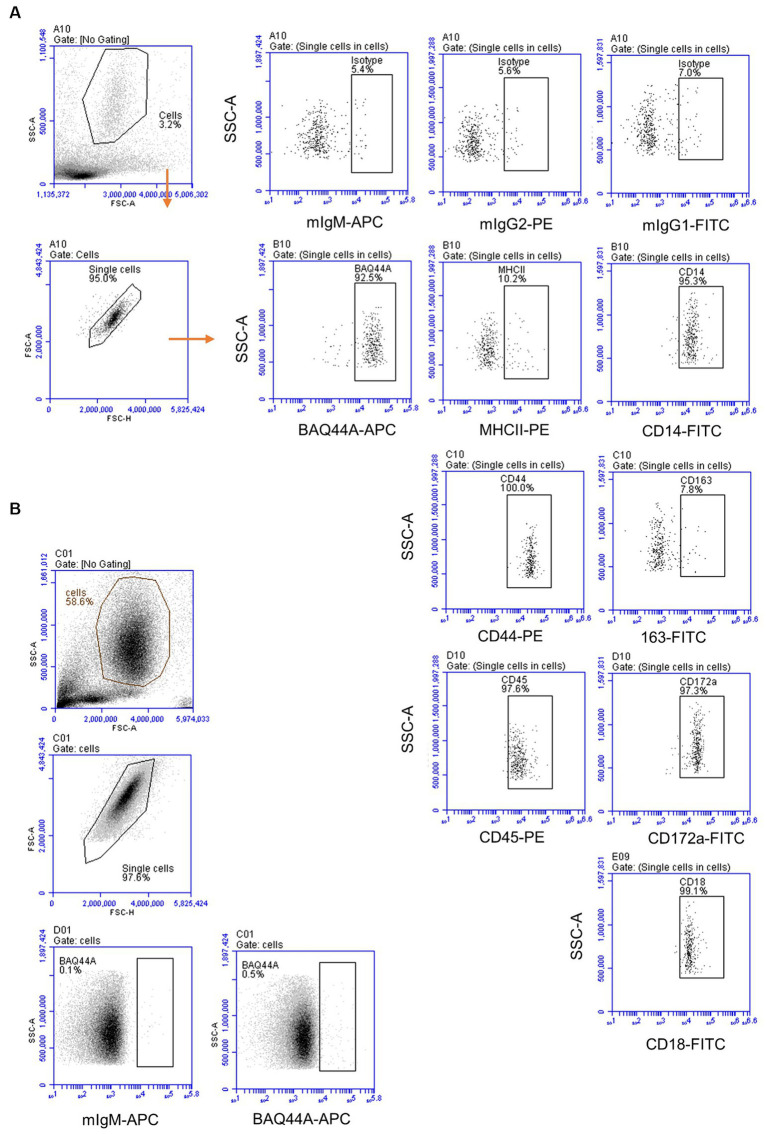
The immunophenotype of lymph node and blood neutrophils. **(A)** Lymph node neutrophils were stained with mAbs to different cell markers and analyzed on the Accuri flow cytometer. **(B)** Blood-derived neutrophils were stained with mAbs to BAQ44A and analyzed by flow cytometry.

The immunophenotype of neutrophils was identified based on their staining with mAbs against CD45, CD172a, CD14, MHCII, CD163, CD44, and CD18 (Figure 6A). Staining with marker-specific mAbs were compared with isotype control background staining. Additionally, the expression of the BAQ44A marker antigen was compared between lymph node (Figure 6A) and blood neutrophils (Figure 6B).

**Figure 7:**
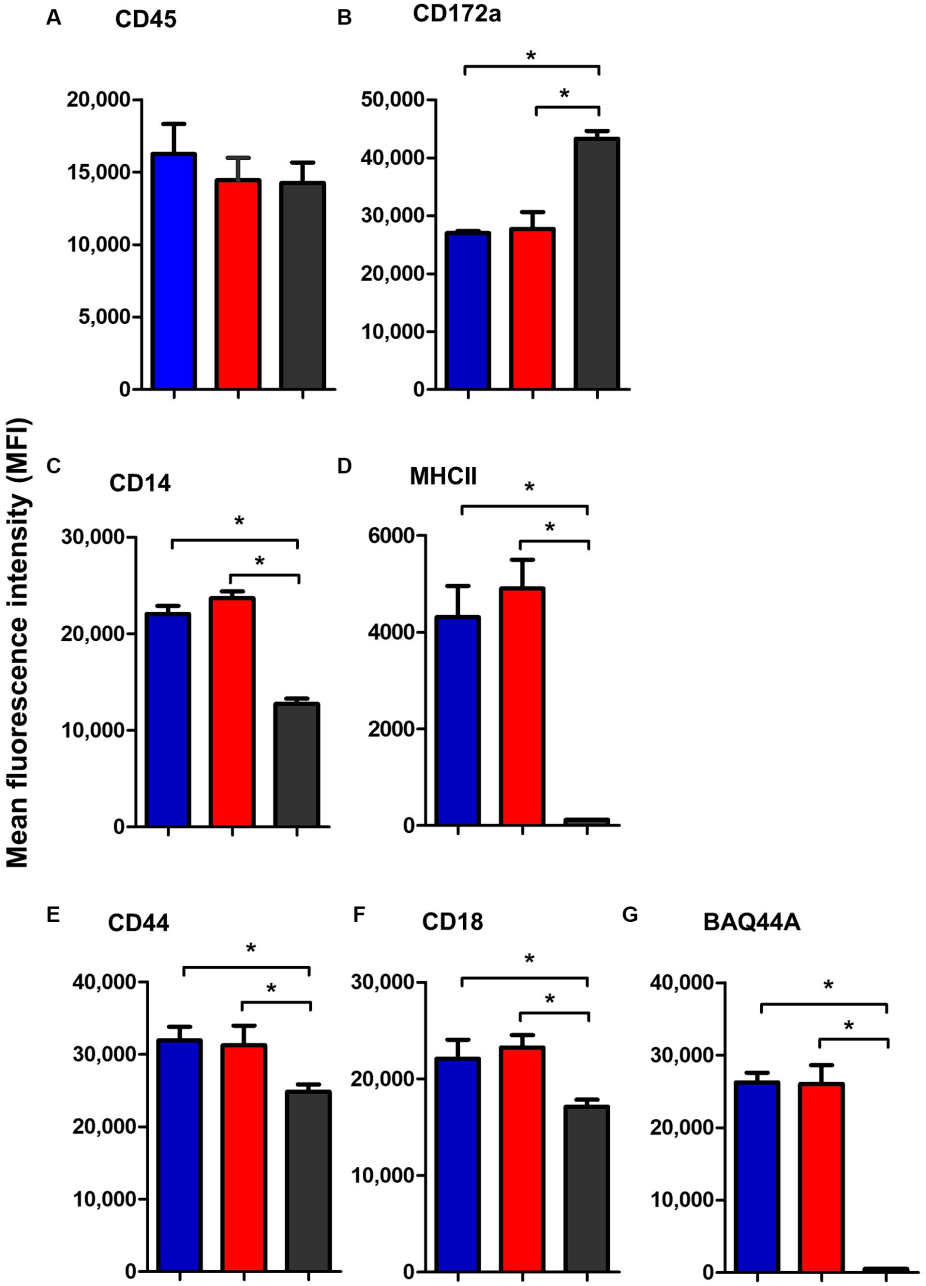
The abundance of CD45 **(A)**, CD172a **(B)**, CD14 **(C)**, MHCII **(D)**, CD44 **(E)**, CD18 **(F)**, and BAQ44A **(G)** on neutrophils from camel bronchial (blue color), mesenteric (red color) lymph node, or blood neutrophils (black color). Molecule expression level was presented as mean fluorescence intensity (MFI) of the corresponding antibody staining.

Similar to blood neutrophils, lymph node neutrophils stained positive for the pan-leukocyte marker CD45, the myeloid marker CD172a, and the LPS-receptor CD14, the cell adhesion molecules CD44 and CD18 with different surface molecules abundance in comparison to blood neutrophils. Compared to their counterparts in blood, lymph node neutrophils showed comparable level of the pan-leukocyte marker CD45 (Figure 7A). The abundance of the monocyte markers CD14 and MHCII (Figures 7C,D) was significantly higher on lymph node cells, while the myeloid marker CD172a was significantly lower expressed on lymph node neutrophils compared to blood neutrophils (Figure 7B). The cell adhesion molecules CD44 and CD18 were also higher expressed on lymph node neutrophils compared to blood neutrophils (Figures 7E,F). Interestingly, lymph node neutrophils showed a positive staining with the B cell marker BAQ44A, which was in contrast to neutrophils from peripheral blood (Figure 7G). For all surface markers, no significant differences could be identified between values for mesenteric and bronchial lymph nodes neutrophils (*p* > 0.05).

## 4 Discussion

Lymphocytes are migratory cells that circulate through the different lymphoid and non-lymphoid organs of the body (17). Although their numbers in the peripheral blood represent only a minor fraction (about 2% in adult human) of the total numbers of lymphocytes pool in the body, the frequency of lymphocyte subsets in the peripheral blood is beneficial in daily clinical routine for the evaluation of the immune status of diseased individuals and to design and monitor treatment plans (18, 19). As changes in composition and phenotype of blood lymphocyte does not always reflect changes in the body lymphocyte compartment, the analysis of the tissue-specific distribution of lymphocyte subsets is of primary importance. Given the limited data on detailed immune cell composition and cell marker expression in the dromedary camel lymph node tissue, the present study was undertaken to investigate the immune cell composition of bronchial and mesenteric lymph nodes from healthy dromedary camels using flow cytometry.

Combined staining with mAbs to the pan-leukocyte marker CD45 and the myeloid markers CD172a and CD14 enabled the differentiation between myeloid and lymphoid cells within camel lymph node cells with a comparable composition to bovine (7) and ovine (20) lymph nodes. In addition, the dominance of CD4+ T cells within camel lymph node lymphocytes seems in line with reports in other ruminants (7, 21, 22). Using the same mAbs clones used in the present study, Navarro et al. found similar percentage of CD4+ T cells in bovine blood and lymph nodes (21). Gamma delta (γδ_) T cells represent a major component of the lymphocyte system in ruminants including camels (23). The workshop cluster (WC) 1 antigen, which is a transmembrane glycoprotein related to the scavenger receptor CD163 (24), was used in the present study to identify camel γδ T cells. Although it is expressed on a large proportion of ruminant γδ T cells, using WC1 mAbs will probably miss WC1-negative γδ T cells (25). Therefore, the development of antibodies to camel γδ TCR is essential for complete identification of all γδ T cell subsets. In the present work, the frequency of MHCII+ and WC1+ lymphocytes in lymph nodes compared to blood indicates a similar tissue distribution pattern of B cells and γδ T cells in camel and bovine (21). All these results support previous reports on the similarity between the camel immune system and the immune system of other ruminants (26). Although mesenteric and bronchial lymph nodes showed similar frequency of lymphoid and monocytic cells as well as of helper and γδ T cell subsets, the lower fraction of B cells in the mesenteric lymph nodes indicates a tissue-specific distribution of these cells in the camel.

The BAQ44A mAb recognizes a non-immunoglobulin 60,000 MW B cell-lineage-specific molecule that has been characterized in ruminants and does not correspond to the human CD nomenclature (27–29). In goats, BAQ44A mAb marked both surface immunoglobulin (sIg)+ and sIg− B cells in the B cell areas in the germinative center dark zone, but did not label plasma cells (30). In the present study, a significant proportion (25 to 40%) of camel peripheral blood B cells (MHCII+CD14− lymphocytes) stained positive with BAQ44A, which is similar to the expression pattern for blood B cells from bovine and small ruminants (21). In contrast, the lower fraction of BAQ44A+ cells within total lymphocytes and MHCII+ lymphocytes in camel lymph nodes cells compared to blood MNC indicates different expression pattern of BAQ44A in camel and other ruminants (30). The functional relevance of this species-specific expression of BAQ44A requires functional characterization of the BAQ44A molecule. Using MHCII staining for the identification of camel B cells represents a limitation of the present study. This is because MHCII molecules could also be detected on a minor subsets of activated T cells in bovine (31) and human (32). In the healthy dromedary camel, although we could not identify any MHCII expression on CD4+ T cells (Supplementary Figure S3), we cannot exclude the induction of such subset within stimulated T cells in diseased animals. Although we tested several antibodies to human and bovine CD19, CD20, CD21 for their reactivity with camel homologous antigens without any potential cross-reactivity, further studies are required to screen antibodies to other specific B cell markers like CD79a and Pax5 for their reactivity with camel B cells.

CD44 is a cell adhesion receptor (also known as P-glycoprotein 1) involved in cell adhesion and migration (33, 34). In immunophenotyping studies involving human and mice, CD44 abundance is usually used as a marker for the differentiation between memory or effector and naïve lymphocytes (4), with low CD44 expression being indicative of the naïve cell phenotype (4, 35). In the present study, lymph node lymphocytes showed lower CD44 expression compared to lymphocytes from peripheral blood, indicating a higher frequency of naïve lymphocytes in the lymph nodes. This was also supported by the lower expression of the cell adhesion molecule CD18 on lymph node compared to blood lymphocytes (36). The presence of naïve T cells in lymphoid and mucosal tissues has been described previously with the evidence that these cells are blood-derived cells that supply the draining lymph nodes with cells that can be rapidly sampled by local antigen-presenting cells (4, 37).

Lymph nodes serve as sites for the immune response against infections, carrying out the crucial role of filtering tissues and tissue fluids. Their immune cell composition and phenotype may reflect pathologies in tissues they drain. Although bronchial and mediastinal lymph nodes are responsible for trapping and immune response to respiratory and intestinal antigens, respectively, recent studies reported the existence of gut-lung axis with a bidirectional migration and cross-talk between immune cells in the two localizations (38, 39). In subsequent *in vivo* investigations, it is plausible to employ the mAbs elucidated in the current study in combination with cell tracking dyes, to examine the homing and migratory behaviors of immune cells within the two compartments in both healthy and diseased states of camels.

For the human and murine systems, recent studies reported tissue-specific phenotype for lymph node circulating neutrophils (10). In the present study, immunostaining of lymph node neutrophils revealed a CD45^++^ CD172a^++^, CD14^+^, MHCII^low^, BAQ44A^+^, CD44^++^, CD18^++^ phenotype with some differences to their phenotype in blood. The expression of low levels of CD14 on lymph node neutrophils seems in line with the expression pattern of this LPS receptor on camel (40) and bovine (41) blood neutrophils. In addition, the lack of BAQ44A expression on lymph node monocytes (Supplementary Figure S4) argues against a general expression of this marker on all lymph node myeloid cells. Uncovering the function of BAQ44A in future studies would help in understanding the relevance of its expression on lymph node neutrophils. In addition, the elevated abundance of the MHCII molecules suggests a role for these tissue cells in antigen presentation and activation of CD4-positive T helper cells in the camel lymph nodes. This finding seems in line with recent reports revealing high MHCII expression on human lymph nodes neutrophils (42, 43). In addition, murine neutrophils gained the ability to activate T helper cells through the up-regulation of MHCII molecules and costimulatory molecules upon stimulation with immune complexes (42, 44). Together, all these findings suggest a new role for neutrophils in antigen presentation and indicate their involvement in the adaptive immune response. In fact, these findings related to the immunophenotype of lymph node neutrophils are still limited by the lack of antibodies to specific neutrophils marker in camel like the CD15 in human, Ly-6G in mice, or CH138A in cattle. As a result, the cell population identified as granulocytes based on their FSC and SSC properties may contain other large cells including stromal cell types with high SSC signals. Therefore, future studies may focus on the isolation of these cells using cell sorting and confirming their phenotype under microscope.

Based on their reactivity with blood and lymph node immune cells, the mAbs used in the current work represent a tool for the analysis of the immune cell composition in camel lymphoid tissues. The expansion of the mAbs toolbox with reactivity to new camel antigens, especially CD8 and NKP46, would help in the identification of other lymphocyte subsets, including camel cytotoxic T cells and NK cells. After the establishment of reference values for the lymphocyte composition in blood and secondary lymphoid organs in healthy animals, subsequent studies may investigate the impact of different physiological (such as age, gender, nutritional status), environmental (stress), and pathological (infectious and noninfectious diseases) factors on the tissue-specific distribution of lymphocyte subsets in camels. The efficacy of the lymph node cell preparation process employed in the current investigation may not uniformly release all node cell populations, thereby accounting for the observed reduced occurrence of CD14+ cells. Nevertheless, the benefit of circumventing the potential impact of digestive enzymes on the cell immunophenotype, as previously documented for the often employed digestive enzymes, Collagenase and Dispase, is noteworthy (45). Another limitation of the present work is the low number of animals used for collection of blood and lymph node samples, which may not be representative of the diversity and variability of the camel population. In addition, the study does not investigate the potential effects of physiological factors, such as age, breed, and gender or environmental factors, such as diet, climate, or living conditions, on the makeup of immune cells, which is another limitation of the present work.

## 5 Conclusion

In conclusion, the present study employed flow cytometric single-cell analysis and monoclonal antibody staining for immunophenotyping the main populations of immune cells in the bronchial and mesenteric camel lymph nodes. A distinct expression pattern was found for the B cell marker BAQ44A in camel lymph node, with only a low fraction of BAQ44A+ cells in camel lymph nodes cells compared to blood MNC. In addition, the lower expression of CD44 and CD18 on lymph node lymphocytes compared to lymphocytes from peripheral blood indicates higher frequency of naïve lymphocytes in the lymph nodes. The identification of key immune cells in the camel lymph node provides a valuable tool to investigate the structure of the camel lymphoid system in health and disease. Further studies are, therefore required to investigate the clinical relevance of the observed results on specific camel respiratory diseases.

## Data availability statement

The raw data supporting the conclusions of this article will be made available by the authors, without undue reservation.

## Ethics statement

The animal studies were approved by Ethics Committee of King Faisal University, Saudi Arabia (KFU-REC-2021-DEC-EA000326). The studies were conducted in accordance with the local legislation and institutional requirements. Written informed consent was obtained from the owners for the participation of their animals in this study.

## Author contributions

JH: Conceptualization, Methodology, Writing – original draft, Writing – review & editing. HA: Conceptualization, Funding acquisition, Methodology, Writing – review & editing. MA-S: Methodology, Writing – review & editing. BF: Methodology, Writing – review & editing. AA: Methodology, Writing – review & editing.
