# Supplementary material for: Flow cytometric analysis of immune cell populations in the bronchial and mesenteric lymph nodes of the dromedary camel
Source: Front Vet Sci. 2024 Apr 30;11:1365319. doi: 10.3389/fvets.2024.1365319 (PMC11091912; doi:10.3389/fvets.2024.1365319)
Supplement: Supplementary file 1 [file Data_Sheet_1.docx]

**Supplementary Figure 1:** Gating strategy for the identification of lymphocytes (A) and monocytes (M) in camel lymph nodes and blood cells. Cells were stained with mAbs to CD45, CD172a and CD14 and analyzed on the flow cytometer. A) After setting a gate on mononuclear cells (based on FSC and SSC characteristics), the percentage of cells stained positive with CD45 mAb was identified within single cells after excluding the cell doublets in a FSC-H against FSC-A density plot. Lymphocytes were identified based on their negative staining with mAbs to CD172a within CD45-positive cells. B) Monocytes were identified within lymph node and blood MNC based on their positive staining with mAbs to CD14 after exclusion of doublets.

**Supplementary Figure 2:** Gating strategy for the identification of lymphocytes based on their FSC and SSC properties. After the exclusion of cell doublets in a FSC-H/FSC-A density plot, the lymphocyte population (Ly) was identifies using a gate on small cells with low granularity. The identified gate was used for the analysis of lymphocyte subsets (Figure 2, 3, and 4).

**Supplementary Figure 3:** Staining of camel blood lymphocytes with antibodies to CD4 and MHCII molecules. After the exclusion of cell doublets in a FSC-H/FSC-A density plot, the lymphocyte population (Ly) was identifies using a gate on small cells with low granularity. Staining of the identified lymphocytes population with antibodies to CD4 and MHCII was shown in a separate density plot. The figure shows CD4+ lymphocytes as MHCII-negative cells.

**Supplementary Figure 4:** Staining of camel blood and lymph node MNC with antibodies to CD14 and BAQ44A molecules. After the exclusion of cell doublets in a FSC-H/FSC-A density plot, the MNC population was identifies based on FSC and SSC signal. Staining MNC with antibodies to CD14 and BAQ44A or isotype control was shown in a separate density plot. The figure shows CD14+ monocytes as BAQ44A-negative cells.
